# Transcriptional landscape of *Mycobacterium tuberculosis* infection in macrophages

**DOI:** 10.1038/s41598-018-24509-6

**Published:** 2018-04-30

**Authors:** Sugata Roy, Sebastian Schmeier, Bogumil Kaczkowski, Erik Arner, Tanvir Alam, Mumin Ozturk, Ousman Tamgue, Suraj P. Parihar, Hideya Kawaji, Masayoshi Itoh, Timo Lassmann, Piero Carninci, Yoshihide Hayashizaki, Alistair R. R. Forrest, Reto Guler, Vladimir B. Bajic, Frank Brombacher, Harukazu Suzuki

**Affiliations:** 1 Division of Genomic Technologies, RIKEN Center for Life Science Technologies, 1-7-22 Suehiro-cho, Tsurumi-ku, Yokohama, 230-0045 Japan; 2 Riken Omics Science Center, 1-7-22 Suehiro-cho, Tsurumi-ku, Yokohama, 230-0045 Japan; 3 Massey University, Institute of Natural and Mathematical Sciences, Auckland, New Zealand; 4 King Abdullah University of Science and Technology (KAUST), Computational Bioscience Research Center (CBRC), Computer, Electrical and Mathematical Sciences and Engineering Division (CEMSE), Thuwal, Saudi Arabia; 5 International Centre for Genetic Engineering and Biotechnology (ICGEB), Cape Town component, Cape Town, 7925 South Africa; 6 University of Cape Town, Institute of Infectious Diseases and Molecular Medicine (IDM), Division of Immunology and South African Medical Research Council (SAMRC) Immunology of Infectious Diseases, Faculty of Health Sciences, University of Cape Town, Cape Town, 7925 South Africa; 7 Riken Preventive Medicine and Diagnosis Innovation Program (PMI), 2-1 Hirosawa, Wako, Saitama, 351-0198 Japan; 8 Present Address: Telethon Kids Institute, The University of Western Australia, 100 Roberts Road, Subiaco, Subiaco, 6008 Western Australia Australia; 9 Present Address: RIKEN Center for Integrative Medical Sciences, 1-7-22 Suehiro-cho, Tsurumi-ku, Yokohama, 230-0045 Japan

## Abstract

*Mycobacterium tuberculosis* (Mtb) infection reveals complex and dynamic host-pathogen interactions, leading to host protection or pathogenesis. Using a unique transcriptome technology (CAGE), we investigated the promoter-based transcriptional landscape of IFNγ (M1) or IL-4/IL-13 (M2) stimulated macrophages during Mtb infection in a time-kinetic manner. Mtb infection widely and drastically altered macrophage-specific gene expression, which is far larger than that of M1 or M2 activations. Gene Ontology enrichment analysis for Mtb-induced differentially expressed genes revealed various terms, related to host-protection and inflammation, enriched in up-regulated genes. On the other hand, terms related to dis-regulation of cellular functions were enriched in down-regulated genes. Differential expression analysis revealed known as well as novel transcription factor genes in Mtb infection, many of them significantly down-regulated. IFNγ or IL-4/IL-13 pre-stimulation induce additional differentially expressed genes in Mtb-infected macrophages. Cluster analysis uncovered significant numbers, prolonging their expressional changes. Furthermore, Mtb infection augmented cytokine-mediated M1 and M2 pre-activations. In addition, we identified unique transcriptional features of Mtb-mediated differentially expressed lncRNAs. In summary we provide a comprehensive in depth gene expression/regulation profile in Mtb-infected macrophages, an important step forward for a better understanding of host-pathogen interaction dynamics in Mtb infection.

## Introduction

Despite the availability of four anti-tubercular drugs and BCG vaccine against *Mycobacterium tuberculosis* (Mtb) infection, tuberculosis still remains one of the most deadly infectious diseases worldwide, claiming over 1.5 million lives globally^1^. It is estimated that one third of the world population is infected with Mtb, however only 5–10% of individuals develop the active tuberculosis disease^2^, whereas the rest remain latently infected during their life time. Therefore defining the immune correlates which leads to host protection or pathogenesis during tuberculosis infection, could lead to the development of new alternative drug treatments^3^. Macrophages regulate inflammation and immune responses to Mtb infection. However, Mtb modulates host immunity by residing and multiplying within lung macrophages^4^. In response to IFNγ which is secreted by T helper 1 cells and natural killer cells, macrophages are polarized to classically activated macrophages (M1 Mph), leading to the secretion of pro-inflammatory mediators, release of reactive oxygen and nitrogen intermediates, inducing protective immune responses against Mtb infection^5,6^. IFNγ stimulation activates IFNγ receptors, Janus kinase, MHC class I and II, guanosine triphosphatases (GTPases), chemokine receptors Cxcl2, Cxcl3, Cxcl4, Cxcl5, immune regulatory transcription factors such as Irf1^7^, Irf8^8^, Batf2^9^, Stat1^10^, Nfκb^11^, Ap1^10^ and many effectors molecules such as TNFα, IL-6, IL-12, TGFβ, IL-10 cytokines and Ccl2, Ccl3, Ccl4, RANTES (Ccl5) chemokines^7,12,13^. Furthermore IFNγ induces phagocyte oxidase and inducible nitric oxide synthase (Nos2) that control Mtb growth by their antimicrobial activities^14,15^. Several studies showed a sharp increase of Th2 cytokines IL-4 and IL-13 which polarize macrophages to an alternative activation status (M2 Mph)^16–18^. During this alternative activation process M2 Mph induces Arginase 1 (Arg1) which is a checkpoint enzyme since it competes with Nos2 for the same substrate L-Arginine. By metabolizing L-Arginine, Arg1 reduces Nitric Oxide (NO) production, tryptophan degradation and T cell proliferation^19–21^. Consequently Mtb has developed a myriad of evasion strategies to escape killing within M1 polarized macrophages by interfering with the macrophage activation status. Once infection is established within macrophages, Mtb is able to down-regulate IL-12 expression in macrophages^22^ and thereby reducing optimal Th1 differentiation and subsequent IFNγ production. Moreover, Mtb blocks the recruitment of NOS2 to the phagosomal membrane, possibly as a means of limiting its exposure to nitric oxide^23^. Once phagocytosed, Mtb employs its prime evasion strategy to interference with intracellular signaling events to establish persistence. Mtb inhibits phagolysosome fusion, thus allowing virulent mycobacteria to persist within an immature phagosomal compartment that shields from the microbicidal challenges activated by the host cell^24,25^. Therefore the interaction between Mtb and M1/M2 macrophage that leads to a drastic genetic or epigenetic level reprogramming is still an area that needs to be further investigated.

Over the last few decades, transcriptional programming of Mtb-infected macrophages has been studied using oligonucleotide microarrays. Gene expression was analyzed from IFNγ-stimulated, live Mtb, heat-killed Mtb, polystyrene beads-stimulated primary macrophages obtained from wild-type, NOS2^−/−^, Phox^−/−^ and NOS2^−/−^Phox^−/−^ which shows that gene induction by Mtb mimicked or synergized with IFNγ-stimulated macrophages^4^. Mtb-infected macrophage-like THP-1 gene profiling using microarray demonstrated an interferon-related signature in transcriptional core response to Mtb infection^26^. Using 858 spot cytokine array from Mtb-infected human monocytes-derived macrophages gene profiling up to 7 days after infection with Mtb showed up-regulation of previously known cytokines^27^. Recently it was also demonstrated that the macrophage transcriptional responses changes depending on Mtb strain infection (CDC1551 expressed higher levels of stress response genes than HN878)^28^. A previous comparative study of gene expression profile of IFNγ or IL-4 stimulated macrophages using microarray showed a delayed and partially diminished response to Mtb in IL-4-stimulated macrophages. The result highlight that IL-4-stimulated alternative macrophages may supports intercellular persistence of Mtb^20^. Of note, large consortia such as ImmGen^29^ and the Human Immunology Genome Project^30^ have contributed immensely by discovering the steady state transcription programs of murine macrophages^31^ and dendrite cells^32^.

Recently the FANTOM5 consortium has generated a comprehensive promoter expression atlas using 953 human and 399 mouse samples^33^, including classical, intermediate, non-classical monocyte samples^34^, which demonstrated promoters for known and novel coding/non-coding transcripts and enhancer expression profile^35^. CAGE (Capped Analysis of Gene Expression) technology was used for this transcriptome analysis and samples were sequenced using single molecule Helicos sequencer (non-biased deepCAGE). The second phase of FANTOM5 further revealed that enhancers reach maximal transcriptional activity prior to promoters in differentiation and activation of mammalian cells^36^. Recently as a satellite study of FANTOM5 we redefined the transcriptional regulatory dynamics of classically and alternatively activated mouse macrophages by identifying novel motifs, TFs, coding and non-coding marker genes using deepCAGE^37^.

Here we focused on the transcriptional landscape of MtbHN878-infected macrophages compared to classical (IFNγ stimulated, M1) and alternative (IL-4/IL-13 stimulated, M2) activation by high throughput transcriptome analysis method, deepCAGE. Further, pre-stimulation with IFNγ or IL-4/IL-13 in Mtb-infected macrophage transcriptome was compared with non-pre-stimulated Mtb-infected macrophages. DeepCAGE analysis allowed us to identify drastic transcriptional reprogramming in Mtb infection and in IFNγ or IL-4/IL-13 pre-stimulation in Mtb infected macrophages. Taken together our CAGE analysis identified novel TFs, non TF genes and lncRNAs therefore redefining the transcriptional landscape of Mtb-infected macrophages. The work is part of Functional Annotation of Mammalian Genome (FANTOM5) project. Data, genomic tools, and co-published manuscripts are summarized online at http://fantom.gsc.riken.jp/5/.

## Results

### Construction of time-course promoter activity profiles for *Mycobacterium tuberculosis*-infected macrophages

**Figure 1:**
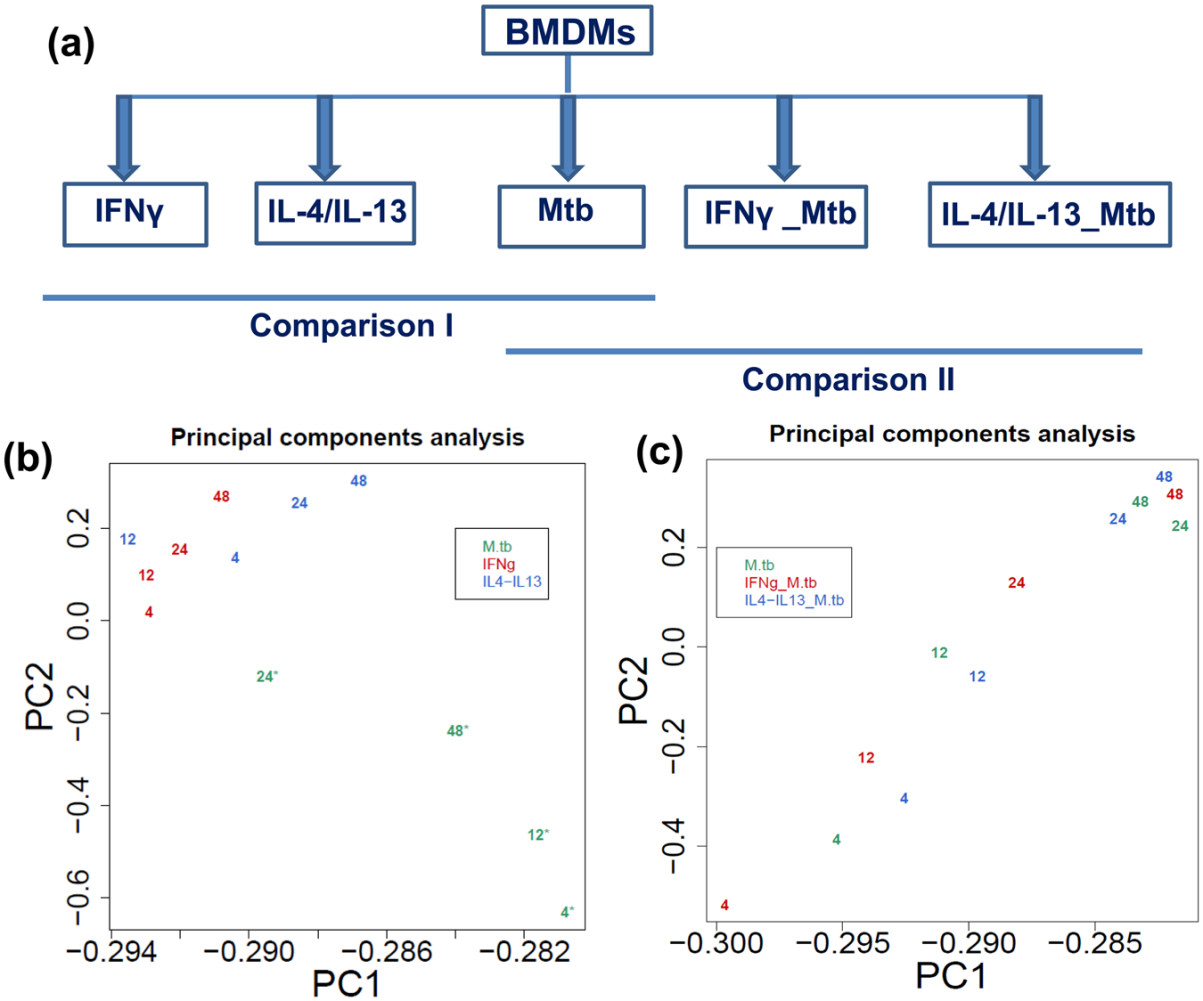
Experimental design and PCA analysis. (**a**) Schematic representation of the experimental design and comparisons to characterize the transcriptional landscape of Mtb-infected macrophages. The first comparison was performed between Mtb-infected macrophage and M1 (IFNγ)- or M2 (IL-4/IL-13)-stimulated BMDMs. The second comparison was performed to analyze the effect of pre-stimulation among Mtb, IFNγ_Mtb and IL-4/IL-13_Mtb. **(b)** Principal component analysis (PCA) was performed between Mtb, IFNγ-treated M1 macrophages, IL-4/IL-13-treated M2 macrophages. **(c)** PCA was performed between Mtb, IFNγ_Mtb and IL-4/IL-13_Mtb stimulated macrophages. Each number in the PCA plots represents the average expression of each sample for the indicated time point. Each condition is depicted with a different color. The PCA analysis using all replicates was shown in Supplementary Fig. 6a and b.

Macrophages are innate immune cells that can be temporally activated to classically (M1) or alternatively (M2) by IFNγ or IL-4 and IL-13, respectively. Macrophages are also the primary (obligatory) target cells for *Mycobacterium tuberculosis* (Mtb) infection. Using *in vitro* cultured primary mouse bone marrow-derived macrophages (BMDMs), we designed and characterized the transcriptional and kinetic (0, 4, 12, 24, 48 hours) landscape of macrophages during Mtb infection using hyper-virulent strain Bejing Mtb HN878. Differentially expressed genes in each condition/time point were compared among M1-and M2-stimulated BMDMs and non-activated Mtb-infected BMDM’s (Fig. 1a, see comparison I). Further, we compared differentially expressed genes of M1- and M2-pre-activated Mtb-infected BMDM’s (IFNγ_Mtb and IL-4/IL-13_Mtb) with those of non-pre-activated Mtb-infected BMDM’s (Fig. 1a, see comparison II), which was designed to explore the differential effects of macrophage M1 and M2 pre-activation in Mtb infection. Total RNA was extracted from harvested cells and subjected to non-amplified deepCAGE transcriptomics using single molecule Helicos sequencers. A flow chart describing the CAGE analysis pipeline was shown for the better understanding of deepCAGE transcriptome analysis (Supplementary Fig. S1). The CAGE tags, computationally mapped on mouse genome (version mm9), ranged from 460,316 to 18,969,698 with a median of 2,655,669 mapped tags among all these libraries (Supplementary Table S1, also http://fantom.gsc.riken.jp/5/). Tags were clustered to identify transcription start site (TSS) regions and create promoter activity profiles (see Materials and Methods). The profiles of three biological replicates showed high correlation in each data point (Supplementary Fig. S2). To verify the quality, we explored the induction of typical and well-known marker genes for M1/M2 activations and Mtb infection; as expected, expression of these marker genes were drastically up-regulated during M1/M2 activation (Supplementary Fig. S3) as well as during Mtb infection (Supplementary Fig. S4), suggesting that both activation and infection were successful. Further, we confirmed the CAGE expression profile of typical marker genes (such as *Tnf*, *Il10*, *Il1b*, *Arg1*) in Mtb, IFNγ_Mtb, IL-4/IL-13_Mtb by qRT-PCR (Supplementary Fig. S5).

For further examining robustness of the genome-wide transcriptomics experiments, promoter level expression data was subjected to the principal component analysis (PCA) in each comparison group (Fig. 1b,c, Supplementary Fig. S6a and b). In comparison I, PCA results suggested that data for Mtb samples were largely separated from IFNγ and IL-4/IL-13 samples during the time kinetic (Fig. 1b and Supplementary Fig. S6a). This suggests that Mtb infection is clearly distinct from macrophage activation, as biologically expected. PCA analysis was also performed for the comparison II among Mtb, IFNγ_Mtb and IL-4/IL-13_Mtb. The results indicate that these three group data were separated in a time dependent manner, with individual time-points of different treatment clustering together (Fig. 1c and Supplementary Fig. S6b). This suggests that differently activated macrophages may react similar during Mtb infection, or that the effects of the much stronger Mtb stimulus are overshadowing the effects of activation.

### Redefining the transcriptional regulatory dynamics of *Mtb-*infected macrophages

In order to characterize the transcriptome of Mtb-infected macrophages, we extracted differentially expressed genes (>2 fold change, FDR < 0.05) at each time point (Table 1 and Supplementary Table S2a and b). Mtb infection drastically increased the number of differentially expressed gene transcripts (>10 fold) in comparison to the transcriptional activity of M1 or M2 activation (Supplementary Table S2c and S2d Table), observed until 48 hours post Mtb infection. In total, 1394 and 915 genes were significantly up- and down-regulated during Mtb infection, respectively. This amounted to 25.4% of the total 9,052 expressed genes of non-stimulated macrophage at 0 h. Of note, Mtb infection also influenced the transcriptional activity of down-regulated genes, particular at 4 and 12 h with 38.3% and 41.7% of differentially expressed genes, respectively, when compared with IFN- or IL-4/IL-13-stimulation at the same time points (11.1~26.3%) (Table 1). For more insights into the differential expression between Mtb infection and macrophage M1 or M2 activation, we explored commonly altered genes. Of interest, IFNγ-induced M1 genes were particularly highly overlapping with Mtb-induced genes with 71 out of 92 (75%), see parenthesis in Table 1. The overlapping genes included *Nos2*, *Tnf*, *Cxcl9*, *Cxcl10*, *Irg1*, among others, consistent with our previous reports (and others) that Mtb infection does induce interferon-related inflammatory responses in macrophages^26,38,39^. Taken together, Mtb infection largely affects macrophage gene expression including inflammatory response genes.

**Table 1:** Number of differentially expressed protein coding genes at each time point

Stimulation	Change	Number of changed genes			
		4 h	12 h	24 h	48 h
Mtb	Up	1262	612	135	80
	Down	783	437	37	29
IFNγ	Up	92 (71)	14 (3)	0	0
	Down	28 (9)	5 (2)	0	0
IL-4/IL-13	Up	26 (11)	8 (2)	7 (1)	4
	Down	5 (1)	1	0	1

IFNγ and IL-4/IL-13 stimulated samples have parenthesis which indicates number of the overlapped genes with Mtb infection at that time point.

**Figure 2:**
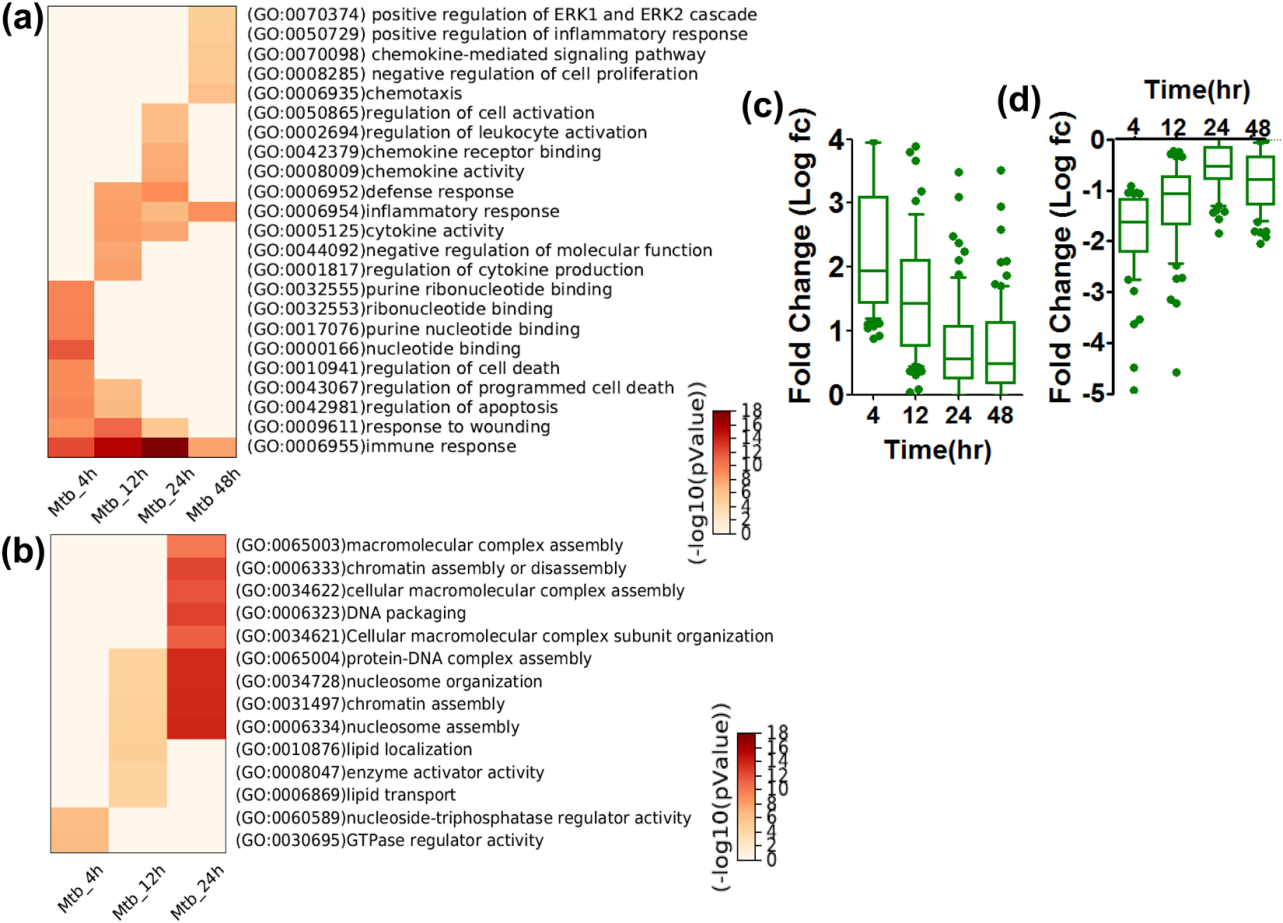
Global effect of Mtb infection in macrophages. (**a**) and (**b**) Heat map of enriched GO terms for up-regulated (**a**) and down-regulated **(b)** genes. Results of top ten gene ontology were shown. Because we did not find significant GO terms for down-regulated genes at 48 h, we showed them up to 24 h. **(c)** and (**d**) Box plot analysis of time course log fold-change expression of differentially up-regulated **(c)** and down-regulated **(d)** TF genes in Mtb-infected macrophages. Boxes show median and interquartile ranges and whiskers show the 10^th^ and 90^th^ percentile values.

To further explore the global effect of Mtb infection in macrophages, differentially expressed genes were subjected to gene ontology analysis at each time point (Fig. 2a,b and Supplementary Table S3a and b). As might be expected from above, Mtb infection induced up-regulated genes revealing significant enrichment of the ontology term “immune response” (GO: 0006955), particularly at 12 and 24 h (Fig. 2a), which was also enriched in IFNγ up-regulated genes at 4 h (Supplementary Table S3c). Most of IFNγ-induced genes in this GO appeared in Mtb infection, although Mtb infection induced additional genes, as demonstrated above. Of immunological significance, several receptors (*Tlr2*, *Tollip*, *Tbk1* and *Cd14*), antioxidant (*Prdx1*), signaling molecules and kinases (*Myd88*, *Nod2*, *Clec4d*, *Clec4e*, *Oasl1*, *Ticam2*, *Bnip3*, *Icam1*), cytokines (*Il10*, *Tnf*, *Il1a*, *Il1b*, *Il12a*, *Il15*, *Il1f9*, *Cfs3*, *Lif*, *Tnfsf9*, *Il6*), chemokines (*Cxcl2*, *Cxcl3*, *Ccl9*, *Ccl4*, *Ccl7* and *Ccl12*) and transcription factor (*Nfκb2*) were only differentially expressed in Mtb infection (Supplementary Table S2a). The ontology term “response to wounding” (GO: 0009611) was also highly enriched for Mtb-up-regulated genes during 4 to 24 h. At earlier time point from 4 and 12 h, apoptosis-related ontology terms, “regulation of apoptosis” (GO: 0042981), “regulation of programmed cell death” (GO: 0043067) and “regulation of cell death” (GO: 0010941), were enriched for the up-regulated genes and disappeared at 24 h. In addition to the above ontology terms, a time-dependent shift of other ontology terms were observed; those were nucleotide binding related terms at 4 h, “nuclectide binding” (GO: 000166), “purinenucleotide binding” (GO: 0017076), “ribonucleotide binding” (GO: 0032553), “purine ribonucleotide binding” (GO: 0032555), and terms related to inflammation regulation at 12 and/or 24 h, “regulation of cytokine production” (GO: 0001817), “negative regulation of molecular function” (GO: 0044092), “cytokine activity” (GO: 0005125), “inflammatory response” (GO: 0006954), “regulation of leukocyte activation” (GO: 0002694), and “regulation of cell activation” (GO: 0050865). Interestingly, term related to inflammation regulation was further enriched at 48 h such as “positive regulation of inflammatory response” (GO: 0050729), “inflammatory response”(GO: 0006954), “negative regulation of cell proliferation” (GO: 0008285), “chemokine-mediated signaling pathway” (GO: 0070098), “positive regulation of ERK1 and ERK2 cascade” (GO: 0070374).

Time-dependent shift of gene ontology terms were also observed for Mtb down-regulated genes (Fig. 2b and Supplementary Table S3b). In particular at the late time point at 24 h, Mtb down-regulated genes were highly significant enrichment for ontology terms such as “nucleosome assembly” (GO: 0006334), “chromatin assembly” (GO: 0031497), “protein–DNA complex assembly” (GO: 0065004), “DNA packaging” (GO: 0006323), which may imply that possible dis-regulation of basic cellular functions may occur in macrophages around 24 h post Mtb-infection. Together, the marked transcriptional regulatory dynamics of *Mtb-*infected macrophages seen might be the result of the cellular war between host immune defense and pathogen evasion responses.

### Differentially expressed TF genes in Mtb infection

The gene expression dynamics during Mtb infection underlays global changes of transcription factor (TF) gene expression. Hence, we explored differentially expressed TF genes in Mtb-infected macrophages. Ninety-nine and sixty TF genes were significantly up- and down- regulated in response to Mtb infection (Supplementary Table S2a and b). Considering 953 TF genes were expressed in BMDM at time 0 h, 16.6% (159/953) of TF gene expression was significantly altered in Mtb infection. The average expression features of up- or down-regulated TF genes revealed a rapid alteration at 4 h and quickly return to the original expression level in the following time point (Fig. 2c,d), consisting with the up- and down-regulated gene expression as this time point.

Next we examined how M1/M2 up-regulated TF genes in this study are affected by Mtb infection. We found that 5 out of 7 M1 up-regulated TF genes (*Batf2*, Irf1, *Stat1*, *Zfp281* and *Zfp800*) and 3 out of 4 M2 up-regulated TF genes (*Bhlhe40*, *Egr2* and *Tfec*) were also up-regulated during Mtb infection (Supplementary Table S2a to d). A literature search showed that Mtb infection up-regulates additional M1 TF genes, including inflammatory/immune TFs (*Crem*, *Arid5a*, *Cebpb, Creb5*, *Jun*, *Fos*, *Nfkb1, Nfkb2* among others) and M2 TF genes (*Myc, Irf4*, *Mafb*, *Ets1*, *Ets2*, *Rel*, *Aft5*, *Batf*, *Fosl2*, *Zc3h12a* among others). However, several M1 TF genes, such as *Irf8*, *Bcl6, Arid4a*, *Pou2f2* etc showed down-regulation. In addition, our data showed that inhibitory/transcriptional repressor TF genes, such as *Id1, Id2 Foxp1, Foxp4, Hivep1, Hivep2, Gabpb1, Gabpa*e and transcriptional repressor genes of *NfκB*, such as *Nfkbia, Nfκbib, Nfκbid, Nfκbie, Nfκbiz* were up-regulated in Mtb infection at early time point. Furthermore, as well as previously reported, TFs *Stat3, Klf6, Klf7, Egr1, Egr2, Etv3, Junb, Rel, Rela, Tfec*, were involved in Mtb infection. In addition, novel up-regulated TF genes such as *Fosl2, Fosl1, Foxp1, Foxp4, Fubp1, Gabpa, Gabpb1, Hivep1, Hivep2, Maff, Mafk, Mafg, Mxd1* among others were identified in our data. Conclusively, Mtb infection perturbs many TFs involved in various macrophage transcriptional regulatory networks including M1/M2 activations, leading to drastic and complex transcriptional reprogramming.

### Pre-stimulation with IFNγ and IL-4/IL-13 showed global effects in transcriptomics of Mtb-infected macrophages

**Figure 3:**
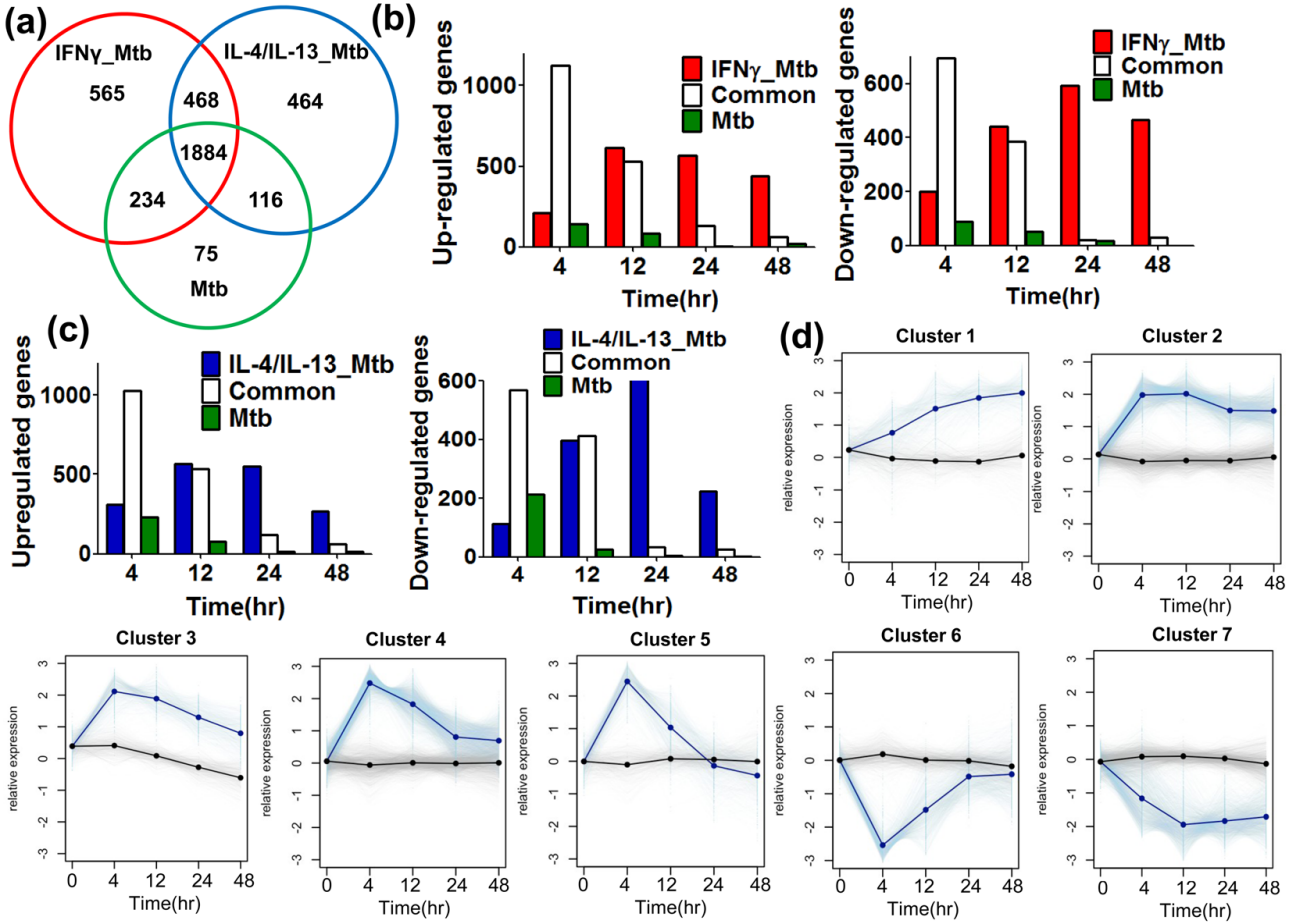
Effect of IFNγ and IL-4/IL-13 pre-stimulation in Mtb-infected macrophages. (**a**) Venn diagram analysis of differentially expressed genes in Mtb, IFNγ_Mtb and IL-4/IL-13_Mtb. The overall landscape of differentially expressed (up and down) genes regardless of time was shown. There are same gene up and down regulated at different time point for the same condition (11 in IFNγ_Mtb and 3 in IL-4/IL-13_Mtb) which were excluded from the venn diagram analysis. (**b**) Number of up-regulated (left panel) and down-regulated (right panel) genes at each time point in Mtb and IFNγ_Mtb. Open column indicates commonly regulated genes and red and green column indicate specifically regulated genes in IFNγ_Mtb and Mtb, respectively. (**c**) Number of up-regulated (left panel) and down-regulated (right panel) genes at each time point in Mtb and IL-4/IL-13_Mtb. Open column indicates commonly regulated genes and blue and green column indicate specifically regulated genes inIL-4/IL-13_Mtb and Mtb, respectively. (**d**) Seven clusters of the *k*-means clustering analysis for differentially expressed genes in Mtb infection are shown. We visualized the time-dependent pattern of expression change by plotting the expression of the genes that were centers of each cluster (thick black line for control and thick blue line for the condition). The remaining genes were plotted in fine lines, grey for control and light blue for the condition. The control is the mean expression of all replicates of unstimulated non-infected BMDM at each time points. Thus the controls average/center around zero (the average fold change of controls vs mean control is zero).

Next, we explored how Mtb infection with IFNγ or IL-4/IL-13 pre-stimulation affects gene expression changes in macrophage (comparison II in Fig. 1a). As shown in the comparison I (Table 1), IFNγ- and IL-4/IL-13-mediated gene expression changes were temporal, as a consequence few differentially expressed genes were observed at 24 h post stimulation, the starting time for Mtb infection. Differentially expressed genes were extracted at each time point after Mtb infection with IFNγ or IL-4/IL-13 pre-stimulation (IFNγ_Mtb or IL-4/IL-13_Mtb, respectively). We obtained 3162 and 2935 differentially expressed genes for IFNγ_Mtb and IL-4/IL-13_Mtb, respectively (Supplementary Table S4a to d). Including the differentially expressed genes in non-stimulated and pre-stimulated Mtb, a total of 3806 differentially expressed genes were found overall. More than half of the genes (1884 genes) were commonly altered in three conditions (see Venn diagram in Fig. 3a). In non-stimulated Mtb-infected macrophages, the number of differentially expressed genes was small (75 genes), while in IFNγ_Mtb or IL-4/IL-13_Mtb we found that the number was several fold higher with 565 and 464 differentially expressed genes, respectively. This suggests that M1 and M2 pre-stimulation promotes distinct and additional differentially expressed genes in Mtb infection. We next explored time kinetics of differentially expressed genes of the three conditions (Fig. 3b,c). The early time point at 4 h, commonly up- and down-regulated genes were dominating, but rapidly decreasing with time, with small number of common genes expressed at 48 h. The differential genes in non-stimulated Mtb-infected macrophages were mainly found in 4 and 12 h post infection, reclining drastically thereafter.

This scenario was very different in up- and down-regulated differentially expressed genes for IFNγ_Mtb and IL-4/IL-13_Mtb. As shown in Fig. 3b in a kinetic expression, up- and down-regulated differential genes in IFNγ_Mtb raised within 12 h to over 500 genes and stayed during the 48 h kinetic. A slightly different dynamic was observed in IL-4/IL-13_Mtb. Indeed, differentially up-regulated genes showed a similar kinetic dynamic as IFNγ_Mtb, raising within 12 h close to over 500 genes and only reclining slowly during the 48 h. Together, this demonstrates that Mtb infection within M1 and M2 has drastic transcriptional changes, compared to Mtb-infected macrophages.

To identify groups of genes that share common patterns of expression change within the duration of the time courses, we clustered the 3806 differentially expressed genes from Fig. 3a by *k*-means clustering. We clustered the differentially expressed genes into 7 clusters based on their expression profile (Fig. 3d). Cluster 1 showed progressive up-regulation, clusters 2, 3 and 7 showed sustained up- and down-regulation during time, and Cluster 4 to 6 transient up- or down-regulation. The number of genes in each cluster shows that Mtb has small populations of progressively up- and sustained down-regulated genes (clusters 1 and 7, respectively) in comparison with IFNγ_Mtb or IL-4/IL-13_Mtb (Supplementary Table S5). The contents analysis revealed that genes involved in clusters 1 and 7 of IFNγ_Mtb and IL-4/IL-13_Mtb consist of higher population of un-differentially expressed genes in Mtb (Table 2). Further, significant population of sustained up-regulated genes in cluster 2 of IFNγ_Mtb and IL-4/IL-13_Mtb were transiently up-regulated genes in cluster 4 of Mtb. Similarly, significant population of sustained down-regulated genes in clusters 7 of IFNγ_Mtb and IL-4/IL-13_Mtb are transiently down-regulated genes in clusters 6 of Mtb. The shifts indicate that pre-stimulation prolonged differential expression in a set of genes. On the other hand, there are few genes that shift opposite direction. Taken together, the results demonstrate global effect that IFNγ_Mtb and IL-4/IL-13_Mtb reveal differentially expressed genes for long time which is in part due to prolonged gene expression change.

**Table 2:** Cluster distribution of differentially expressed genes between Mtb and IFNγ_Mtb (A) and between Mtb andIL-4/IL-13_Mtb (B).

		Mtb							
		Cluster 1	Cluster 2	Cluster 3	Cluster 4	Cluster 5	Cluster 6	Cluster 7	Un-differentially expressed
IFNγ_Mtb (A)	Cluster 1	24	23	1	0	0	0	0	131
	Cluster 2	5	228	32	153	11	0	0	131
	Cluster 3	1	16	104	22	21	0	0	86
	Cluster 4	0	60	7	365	51	0	0	94
	Cluster 5	0	0	5	74	65	0	0	80
	Cluster 6	0	0	0	0	0	435	16	200
	Cluster 7	0	0	0	0	2	237	148	334
IL4/IL13_Mtb (B)	Cluster 1	27	26	7	1	0	1	0	149
	Cluster 2	7	250	11	154	5	0	0	231
	Cluster 3	0	12	104	16	1	0	0	44
	Cluster 4	0	36	5	323	46	0	1	103
	Cluster 5	0	0	8	73	75	0	0	56
	Cluster 6	1	0	0	0	0	363	6	86
	Cluster 7	0	0	0	0	5	262	163	277

### Mtb infection augmented cytokine-mediated M1 and M2 activations

**Figure 4:**
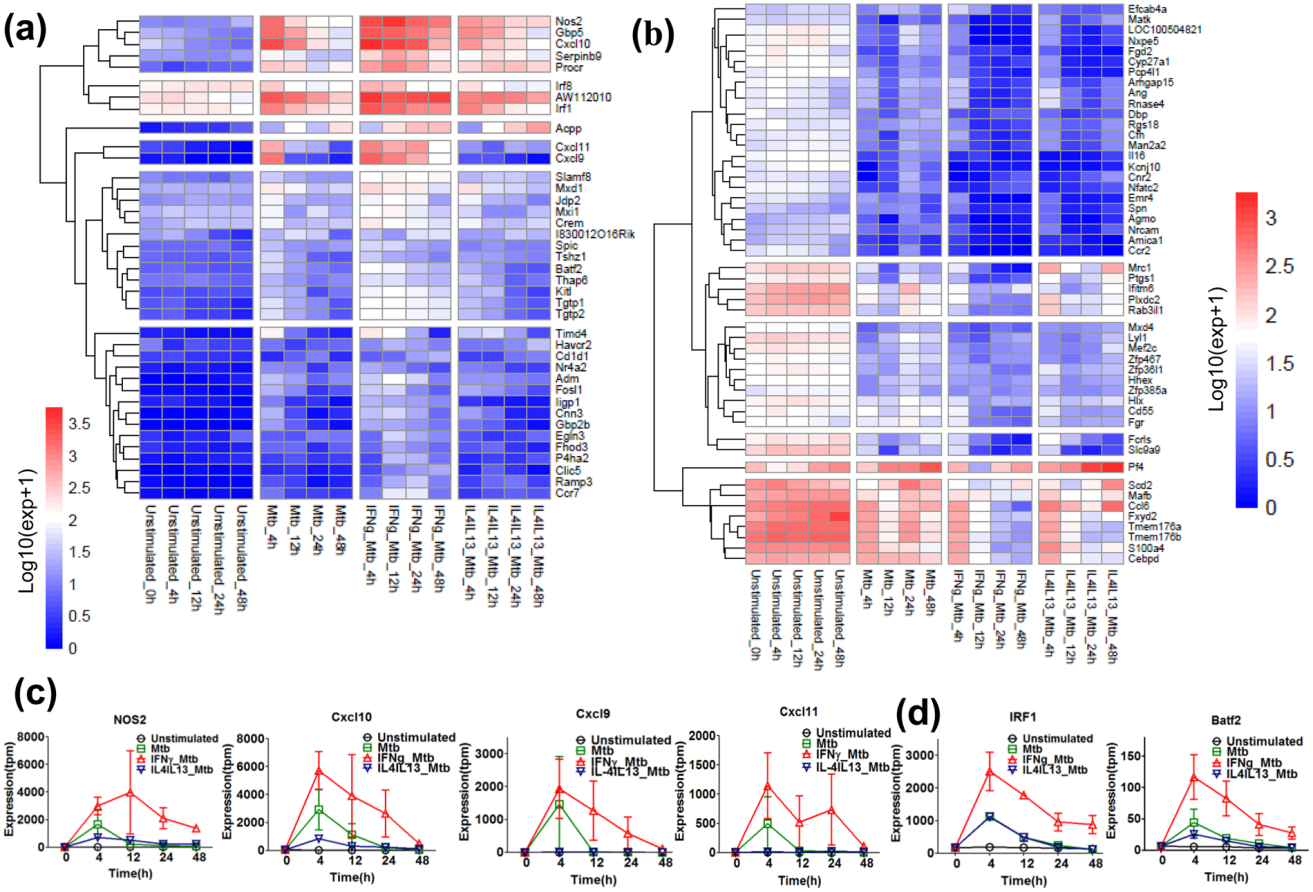
Mtb infection augments M1 gene activation in M1 pre-activated macrophages. Using the differential expressed genes, qualitative analysis was performed in Mtb, IFNγ_Mtb, IL-4/IL-13_Mtb samples. Several fold elevated or suppressed genes were identified by calculating TMP expression fold change of IFNγ_Mtb in comparison with Mtb, IL-4/IL-13_Mtb samples at each time point. IFNγ pre-stimulation-mediated several fold up regulated **(a)** and suppressed or down regulated **(b)** non TF genes (10-fold respectively) and TF genes (3-fold respectively) was selected as seen in the heatmap cluster in each time point. Both heatmaps shows un-stimulated control which indicate the basal level of gene expression. **(c)** Expression profiles of representative M1 key effector genes, *Nos2*, *Cxcl10*, *Cxcl9* and *Cxcl11*. **(d)** Expression profiles of representative M1 key TF genes, *Batf2* and *Irf1*.

To explore Mtb-induced differentially expressed genes that are drastically affected in pre-stimulation-specific manner, we selected pre-stimulation-mediated elevated and suppressed non TF genes (more or less than 10-fold) and TF genes (more or less than 3-fold) in IFNγ_Mtb (Fig. 4a,b and Supplementary Table S6a to S6d). We found that many of the well characterized IFNγ-induced M1 effectors, such as *Nos2*, *Cxcl10*, *Cxcl9*, *Cxcl11*, *Gbp5*, *Timd4*, *Tgtp1*, *Tgtp2*, *Serpinb9* were drastically enhanced by IFNγ pre-stimulation (Fig. 4a). Interestingly, *Cxcl10*, *Cxcl9*, *Cxcl11* and particularly *Nos2*, which subsequently protect macrophages from mycobacterial infection, were drastically enhanced and continuously expressed till 48 h in IFNγ_Mtb (Fig. 4c). These transcriptional reprogramming seemed to be regulated by striking induced and prolonged M1 key TFs, such as *Batf2, Irf1*, *Irf8*, *Crem*, *Fosl1*, *Jdp2*, *Nr4a2* along with novel TFs *Mxi1*, *Mxd1*, *Spic*, *Tshz1* (Fig. 4a). Recently, we disclosed that Batf2, together with Irf1, plays a crucial role of inflammatory gene induction. Expression of *Batf2* and *Irf1* was enhanced in IFNγ_Mtb, which is consistent with induction of their downstream genes (Fig. 4d). Further, IFNγ pre-stimulation drastically down-regulated the expression of several well established M2 marker genes such as*Mrc1*, *Ptgs1*, *Pf4* and *Mafb*, indicating that M1 pre-activation inhibits alternative macrophage activation in Mtb infection (Fig. 4b).

**Figure 5:**
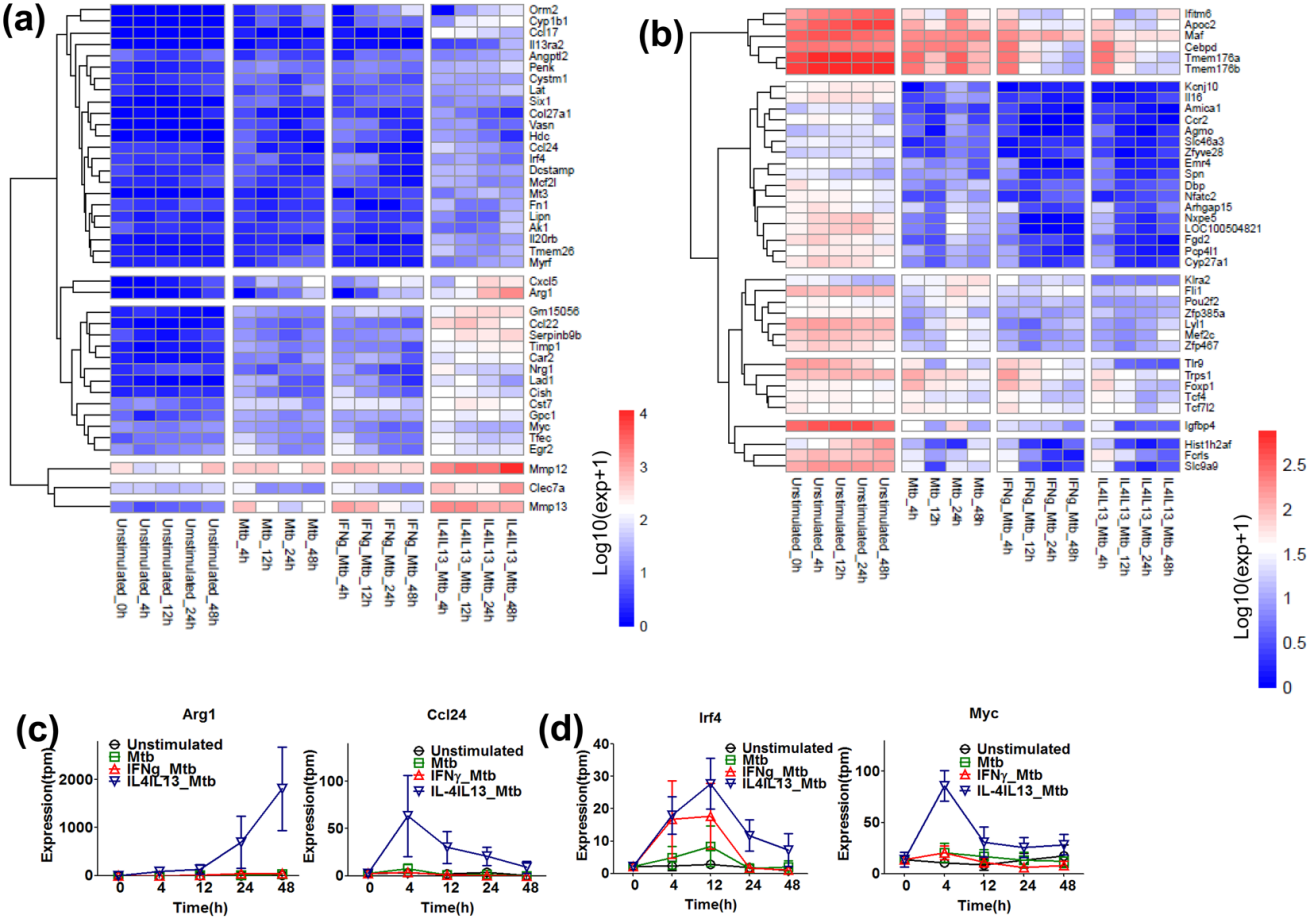
Mtb infection augments M2 activation in M2 pre-activated macrophages. Qualitative analysis was performed to obtain several fold elevated or suppressed genes in IL-4/IL-13_Mtb by calculating TPM expression fold change comparing expression TPM value of IL-4/IL-13_Mtb with Mtb, IFNγ_Mtb samples at each time point. IL-4/IL-13 pre-stimulation-mediated several fold up regulated **(a)** and suppressed or down regulated **(b)** non TF genes (more and less than 10-fold respectively) and TF genes (more and less than 3-fold respectively) was selected as seen in the heatmap cluster in each time point. Both heat maps shows un-stimulated control which indicate the basal level of gene expression. (**c)** Expression profiles of representative M2 key effector genes, *Arg1* and *Ccl24*. **(d)** Expression profiles of representative M2 key TF genes, *Irf4* and *Myc*.

On the other hand, Mtb infection with pre-stimulation by IL4/IL13 drastically induced many well characterized M2 marker genes such as *Arg1*, *Ccl24*, *Ccl22*, *Ccl17*, *Cxcl5*, *Mrc1*, *Fn1*, *Cish*, *Mmp12*, including recently identified M2 marker genes by our group, such as *Lad1*, *Car2*, *Lipn*, *Gm15056*, *Il20rb*, *Nrg1*, *Serpinb9b, Angptl2*, *Timem26*, which were up-regulated and continuously expressed (Fig. 5a,c). We also found enhancement of well-known M2 key TF gene, such as *Irf4*, *Myc*, *Tfec*, *Egr2* along with two novel TF genes *Six1* and *Myrf*, which might be responsible for the transcriptional shift in IL4/IL13_Mtb (Fig. 5a,d). Recently, *Tlr9* was identified as involved in Mtb infection was down-regulated by IL4/IL13 pre-stimulation. Interestingly, *IL16* and *Ifitm6*, which function as a pro-inflammatory and anti-inflammatory genes in macrophage were down-regulated by IFNγ and IL4/IL13 pre-stimulation (Figs 4b and 5b, respectively). Of note, IFNγ and IL4/IL13 pre-stimulation drastically down-regulated non-TF genes such as *Fcrls*, *Arhgap15*, *Pcp4l1*, *Tmem176a, Tmem176b*, *Emr4*, *Nxpe5, Slc9c9*, *Amica1*, *Fgd2*, *Kcnj10*, *Cyp27a1*, and TF gene such as *Nfatc2*, *Lyl1, Dbp*, *Cebpd*, irrespective of classical or alternative activation in Mtb infection (Figs 4b and 5b).

Finally, we carried out gene ontology enrichment analysis using exclusively differentially expressed genes for IFNγ_Mtb or IL-4/IL-13_Mtb (565 and 464 in Fig. 3a, respectively). We found that another set of genes involved in the ontology term “immune system process” (GO: 0002376), “innate immune response” (GO: 0045087) are enriched in time points of 4, 24 and 48 h in IFNγ_Mtb exclusively up-regulated genes and the ontology, “nucleosome assembly” (GO: 0006334), “chromatin silencing” (GO: 0006342), “regulation of gene silencing” (GO: 0060968), “DNA methylation on cytosine” (GO: 0032776), “DNA replication-dependent nucleosome assembly” (GO: 0006335) in later time point of 48 h in IL-4/IL-13_Mtb exclusively down-regulated genes (Supplementary Table S3d and e). These results indicate that IFNγ or IL4/IL13 pre-stimulation in Mtb infection augments and prolongs M1 or M2 activations, respectively.

### Differentially expressed lncRNAs in Mtb-infected macrophages

**Figure 6:**
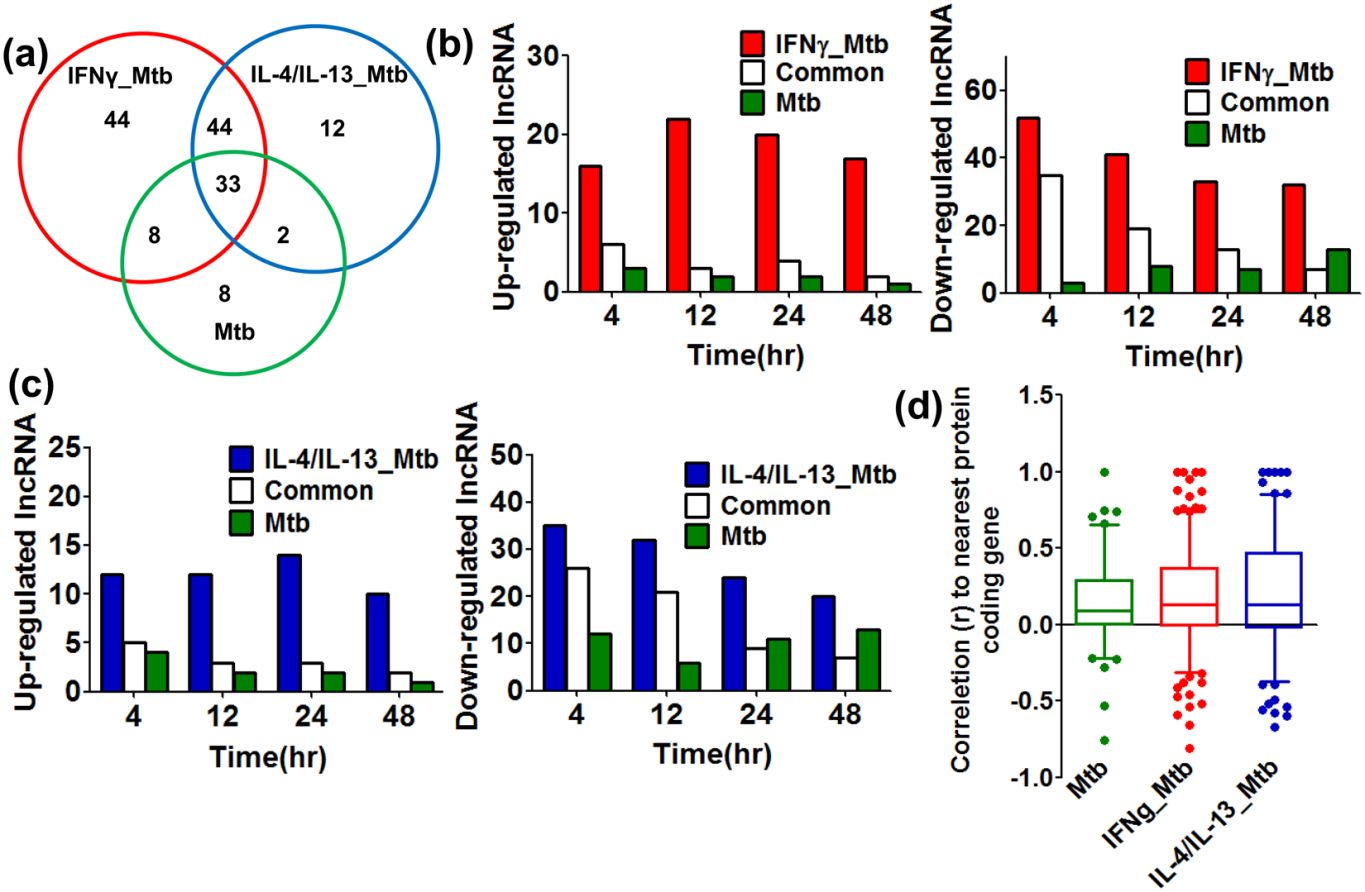
Differentially expressed lncRNAs in Mtb infected macrophages shows unique transcriptional features. (**a**) Venn diagram analysis of differentially expressed lncRNAs in Mtb, IFNγ_Mtb and IL-4/IL-13_Mtb. The overall landscape of differentially expressed (up and down) genes regardless of time was shown. (**b**) Number of up-regulated (left panel) and down-regulated (right panel) lncRNAs at each time point in Mtb and IFNγ_Mtb. Open column indicates commonly regulated lncRNAs and red and green column indicate specifically regulated lncRNAs in IFNγ_Mtb and Mtb, respectively. **(c)** Number of up-regulated (left panel) and down-regulated (right panel) lncRNAs at each time point in Mtb andIL-4/IL-13_Mtb. Open column indicates commonly regulated lncRNAs and blue and green column indicate specifically regulated lncRNAs in IL-4/IL-13_Mtb and Mtb, respectively. **(d**) Expression correlation between differentially expressed lncRNAs and their nearest protein coding genes. Boxes show median and interquartile ranges and whiskers show the 10^th^ and 90^th^ percentile values.

Long non-coding RNAs (lncRNAs) play important roles of regulation of gene expression in various ways, although the function of majority of lncRNAs is unknown. We explored differentially expressed lncRNAs at each time point in Mtb infection. We obtained a total 151 differentially expressed lncRNAs species, as detailed for Mtb (51), IFNγ_Mtb (129) or IL-4/IL-13_Mtb (91) (Fig. 6a and Supplementary Table S7a to c). It is evident that pre-stimulations distinctly enhanced the number of differentially expressed lncRNA, as well as protein-coding genes, although higher number of lncRNAs were differentially expressed in IFNγ_Mtb (129/151 = 85.4%) in comparison with IL-4/IL-13_Mtb (91/151 = 60.3%). Interestingly, Venn diagram analysis revealed that only 33 out of 151 totally differentially expressed lncRNAs (21.9%) were commonly altered (Fig. 6a), which is in contrast with 1884 out of 3806 (49.5%) in protein-coding genes (Fig. 3a). Another interesting feature was that the majority of differentially expressed lncRNAs were down-regulated (42/51 = 82.3%, 96/129 = 74.4% and 69/91 = 75.8%) in Mtb, IFNγ_Mtb and IL-4/IL-13_Mtb, respectively) (Supplementary Table S7). This is in contrast with that major differential expression of protein coding genes were up-regulated in Mtb infections and M1 or M2 activations (Table 1). Next we explored the number of differentially expressed lncRNAs at each time point (Fig. 6b,c). The number slightly decreased within the three conditions, as there was no drastic transient up-regulation nor down-regulation in differentially expressed lncRNAs, which was in contrast with those in protein-coding genes (Fig. 3b,c).

We also investigated expression correlation between differentially expressed lncRNAs and their nearest protein coding genes (Fig. 6d and Supplementary Table S7a to c). It became evident that there is a shift to positive correlation on average in Mtb-infected samples, either with or without M1 or M2 pre-activations, although each correlation value is variable (Fig. 6d). Interestingly, number of lncRNAs, with their expression extreme positive correlation (r > 0.90) with that of their nearest genes, was only 1 for Mtb but drastically increased to 5 for IFNγ_Mtb and 6 for IL-4/IL-13_Mtb (Supplementary Table S7a to c).

Conclusively, Mtb infection in M1 as well as in M2 drastically induced lncRNAs compared to unstimulated macrophages.

## Discussion

In this study, we comprehensively analyzed the transcriptome of Mtb-infected macrophages and the effect of IFNγ (M1) or IL-4/IL-13 (M2) pre-stimulation in a time-dependent manner using our unique CAGE technology. Our results showed that Mtb infection widely and drastically altered macrophage gene expression including induction of inflammatory response genes, which is far larger than that of M1 or M2 macrophage activation. This drastic gene expression change was mediated by expressional alteration of various known and novel Mtb-infection related TF genes including both M1 and M2 activation. We also showed that M1 or M2 pre-activation induces global effect on transcriptional landscape of Mtb-infected macrophages by augmenting M1 or M2 genes and promoting additional genes. Furthermore, we described distinct features of lncRNA expression in Mtb-infection.

Mtb-induced alteration of large number of macrophage gene expression has already been reported in several previous studies^4^. We extended this field in detail by utilizing a kinetic Mtb infection approach. One of the major role of macrophages is to produce inflammatory response, processing of antigen through MHC class I molecule and to engage in pathogen killing, which is regulated by M1 activation^13,40^. However, GO enrichment analysis of Mtb-induced up-regulated genes revealed that not only M1-related immune/inflammation response genes but also M2 genes related to cell wounding and apoptosis are involved. Actually, many of both M1 and M2 TF genes were up-regulated in Mtb infection. Thus, this Mtb-mediated wide range response may be partly responsible for alteration of large number of gene expression in macrophages. We also observed high population of Mtb-induced down-regulated genes, which is consistent with relatively high number of down-regulated TF genes. Because down-regulated genes are rare in M1 or M2 activation, this observation is one of characteristic in Mtb infection. Actually, GO terms related to dis-regulation of basic cellular functions are enriched in the down-regulated genes. However, it is elusive whether those down-regulated genes are pathogenic effect of Mtb infection, since it may be a side reaction that macrophages converge host-protective response.

We showed that M1 or M2 pre-activation in Mtb-infected macrophages augmented M1 or M2 effector genes, respectively. Although molecular mechanism how the augmentation occurs remains to be elucidated, this reaction is interesting from the view of host protection and subversiveness. Mtb infection in M1 pre-activated macrophages enhances expression of inflammatory genes, which seems a reasonable host-protective reaction^4,26^. On the other hand, because Mtb favors to reside within M2 polarized macrophages^20^, augmentation of M2 effector genes in Mtb-infected M2 pre-activated macrophages is considered to be host subversive. The later reaction may be host evasion mechanisms employed by Mtb to establish its persistence and survival within M2 polarized macrophages.

We also successfully identified differentially expressed lncRNAs in Mtb-infected macrophages. Although M1 or M2 pre-activation enhanced altered lncRNAs as well as coding transcripts, the number of commonly altered lncRNAs was not high and the majority of the alteration was indeed down-regulation^41,42^. We showed in a previous report that majority of M1- or M2-mediated differentially expressed lncRNAs was up-regulation^37^. Thus, unique feature of altered lncRNAs in Mtb infection may have significance. Recently, many lncRNAs have been demonstrated to play a role of transcriptional regulation of their neighbor genes^43^. In fact, we detected positive correlation of expressional change between altered lncRNAs and their neighbor genes (Fig. 6d). These functional perturbation analysis of newly identified lncRNAs should enable us for a deeper and better understanding of the role of these transcripts in Mtb-infected macrophages.

This comprehensive promoter-based transcriptome data of Mtb-infected macrophages, together with previously reported data, will be a valuable resource for the research community, particular in immunology, for gaining new insights into Mtb host evasion. Newly identified protein-coding and noncoding transcripts, which altered in Mtb infection in the presence and absence of M1 or M2 pre-activation, may serve as potential transcriptional biomarkers of Mtb infection. Further, those altered transcripts could be a potential source, leading to host-directed Mtb drugs, as part of them may be regulated by Mtb to be host subversive, and may be resistant to drug tolerance and prolonging current Mtb drugs.

## Materials and Methods

### Generation of bone marrow-derived macrophages

BALB/c mice were purchased from Jackson Laboratories and bred in South Africa. Bone marrow-derived macrophages (BMDMs) were generated from 8–12 week old male BALB/c mice as described previously^37^.

### Ethics Statement

Mice were sacrificed in accordance with the Animal Research Ethics of South African National Standard (SANS 10386:2008) and University of Cape Town of practice for laboratory animal procedures. The protocol (Permit Number: 012/036) was approved by the Animal Ethics Committee, Faculty of Health Sciences, University of Cape Town, Cape Town, South Africa.

### Stimulation with IFNγ or IL-4/IL-13 and Mtb infection

BMDMs were plated in 6-well plates (Nunc, Denmark) at 2 × 10^6^ cells per well for adherence. Next morning cells were either left untreated or stimulated with IFNγ (100 unit/ml, BD Biosciences, San Jose, CA) or IL-4/IL-13 (100 units/ml each, BD Biosciences, San Jose, CA) and incubated at 37^o^C under 5% CO_2._ At 0, 4, 12, 24 and 48 hours post stimulation, cells were harvested, lysed with 700 μl of Qiazol (Qiagen, Valencia, CA, USA). For Mtb infection, 24 hours pre-stimulated and untreated BMDMs were infection with log phase *Mycobacterium tuberculosis* HN878 (MOI = 5) for 4 hours. Cells were washed to remove extracellular mycobacteria and replenished with fresh medium containing IFNγ or IL-4/IL-13 and 10 µg/ml of gentamycin. At 4, 12, 24 and 48 hours post infection, cells were harvested. Cell viability was checked (Supplementary Fig. S7). Cells were treated as mentioned above with Qiazol and stored at minus 80^o^C for RNA extraction. All Mtb infection experiments were performed at the Biosafety Level 3 (BSL-3) laboratory, Faculty of Health Sciences, University of Cape Town. Town Total RNA was prepared using miRNAeasy kit (Qiagen, Valencia, CA, USA) and concentration and quality of each RNA samples was checked as described previously^37^.

### Preparation of Helicos CAGE library, sequencing

Single molecule Helicos CAGE library was prepared, sequenced, mapped and clustered into TSS regions as described previously^36,37^. Briefly, each library was prepared by using 5 μg of total RNA, with RIN value of more than 7.5 (Supplementary Table S1). Three to four biological replicates were prepared per time point.

### Construction of promoter data

To identify peaks (TSS clusters) in the CAGE profiles, decomposition peak identification (DPI) was used as described previously^36^. To calculate normalization factors for the expression of promoters we used “relative log expression (RLE)”^44^ method as described previously^33,37^. Reproducibility between replicates was assessed by computing pair wise Pearson correlations between samples across TSS regions after addition of a pseudo count of one and logarithmic transformation (Supplementary Fig. S2).

### Principal component analysis

Principal component analysis (PCA) was performed using the R-function ‘prcomp’ on a TSS level. TSS regions of triplicates were averaged prior to PCA. We only considered TSS regions with a minimum of 5 tags in at least one sample^37^.

### Differential expression analysis and qualitative analysis of protein-coding genes

Reads mapped to DPI/promoter regions were counted and used as input to perform differential expression (DE) analysis as described previously using edgeR^44,45^. IFNγ- and IL-4/IL-13-stimulated BMDM samples at time-points 4, 12, 24, and 48 h post-stimulation were compared to unstimulated BMDM samples at the same time-points, except for 4 and 12 h time-points, which were compared to unstimulated 0 h samples as described previously. Furthermore, Mtb infected (Mtb), IFNγ or IL4/IL13 pre-stimulated and Mtb infected (IFNγ_Mtb or IL-4/IL-13_Mtb) samples at time-points 4, 12, 24, 48 h were compared with unstimulated samples of each time points. Differentially expressed genes were selected using an FDR < 0.05 and a log2 fold-change >1 (log2 fold <−1 in case of down-regulation) in each comparison. Finally, we filtered for robustly expressed DE genes using a 20 tags-per-million (TPM) threshold in the respective comparison. We also created differentially expressed genes for IFNγ_Mtb or IL-4/IL-13_Mtb by comparing with IFNγ or IL-4/IL-13 pre-activated BMDM, resulting in similar differentially expressed genes with the above comparison. Therefore we did not use this comparison for our analysis.

The qualitative analysis was performed using DE gene data of Mtb, IFNγ_Mtb, IL-4/IL-13_Mtb samples. Gene expression up- or down-regulation was calculated based on TPM expression fold-changes between Mtb, IFNγ_Mtb, and IL-4/IL-13_Mtb samples. Several-fold elevated or suppressed genes were identified by comparing the expression values of IFNγ_Mtb with Mtb, IL-4/IL-13_Mtb samples. Similarly, several-fold elevated or suppressed genes in IL-4/IL-13_Mtb samples were identified by comparing them with Mtb, IFNγ_Mtb samples. We selected non-TF genes (more or less than 10-fold expressed) and TF genes (more or less than 3-fold expressed) among all significantly differentially expressed genes.

### Differential expression analysis of lncRNA promoters

Differential expression analysis of lncRNAs was performed by counting all mapped reads within the DPI/promoter regions as described previously using edgeR^44^. We subselected transcripts that had at least 1 TPM expression in at least two replicates in each sample groups. Differentially expressed lncRNA promoters were selected using FDR < 0.05 (Benjamini-Hochberg FDR) and a log2 fold-change >1 (log2 fold <−1 in case of down-regulation) in each comparison.

### Gene ontology analysis

Gene ontology analysis was performed using the database for Annotation, Visualization and integrated discovery (DAVID). The top gene ontology terms were selected using p value of 0.001 and FDR 0.05.

### *K*-means clustering analysis

We performed *k-*means clustering to identify groups of genes that share common patterns of expression change within the duration of the time course. First, for each time-course experiment we extracted the normalized expression values of differentially expressed genes. The triplicates were averaged into one expression profile. We then merged the expression tables from 3 time-courses, thus a given gene is represented in each time-course in which it was found differentially expressed. In total, we obtained 8,426 expression profiles (since most of the genes were differentially expressed in more than 1 condition). The data were log10 transformed, after adding an offset of 0.1. Subsequently, for each gene we scaled the data to the mean expression of 0 within the control time points and to standard deviation of 1. This way we calculated the expression values that were relative to the average control time points, which allowed us to cluster the time-dependent response pattern relative to the baseline. We then clustered the 8,426 expression profiles into 7 clusters using *k-*means algorithm as implemented in *R* (seed fixed at: 123, default settings).

## Electronic supplementary material

Supplementary Information

Supplementary Table S1

Supplementary Table S2

Supplementary Table S3

Supplementary Table S4

Supplementary Table S6

Supplementary Table S7
