# Supplementary material for: Transcriptional landscape of Mycobacterium tuberculosis infection in macrophages
Source: Sci Rep. 2018 Apr 30;8:6758. doi: 10.1038/s41598-018-24509-6 (PMC5928056; doi:10.1038/s41598-018-24509-6)
Supplement: Supplementary file 1 — Supplementary Information [file 41598_2018_24509_MOESM1_ESM.pdf]

Supplementary Information

Transcriptional landscape of *Mycobacterium tuberculosis* infection in macrophages

Sugata Roy, Sebastian Schmeier, Bogumil Kaczkowski, Erik Arner, Tanvir Alam, Mumin Ozturk, Ousman Tamgue, Suraj P. Parihar, Hideya Kawaji, Masayoshi Itoh, Timo Lassmann, Piero Carninci, Yoshihide Hayashizaki, Alistair R. R. Forrest, Reto Guler,Vladimir B.Bajic,Frank Brombacher, Harukazu Suzuki

Supplementary Figure S1

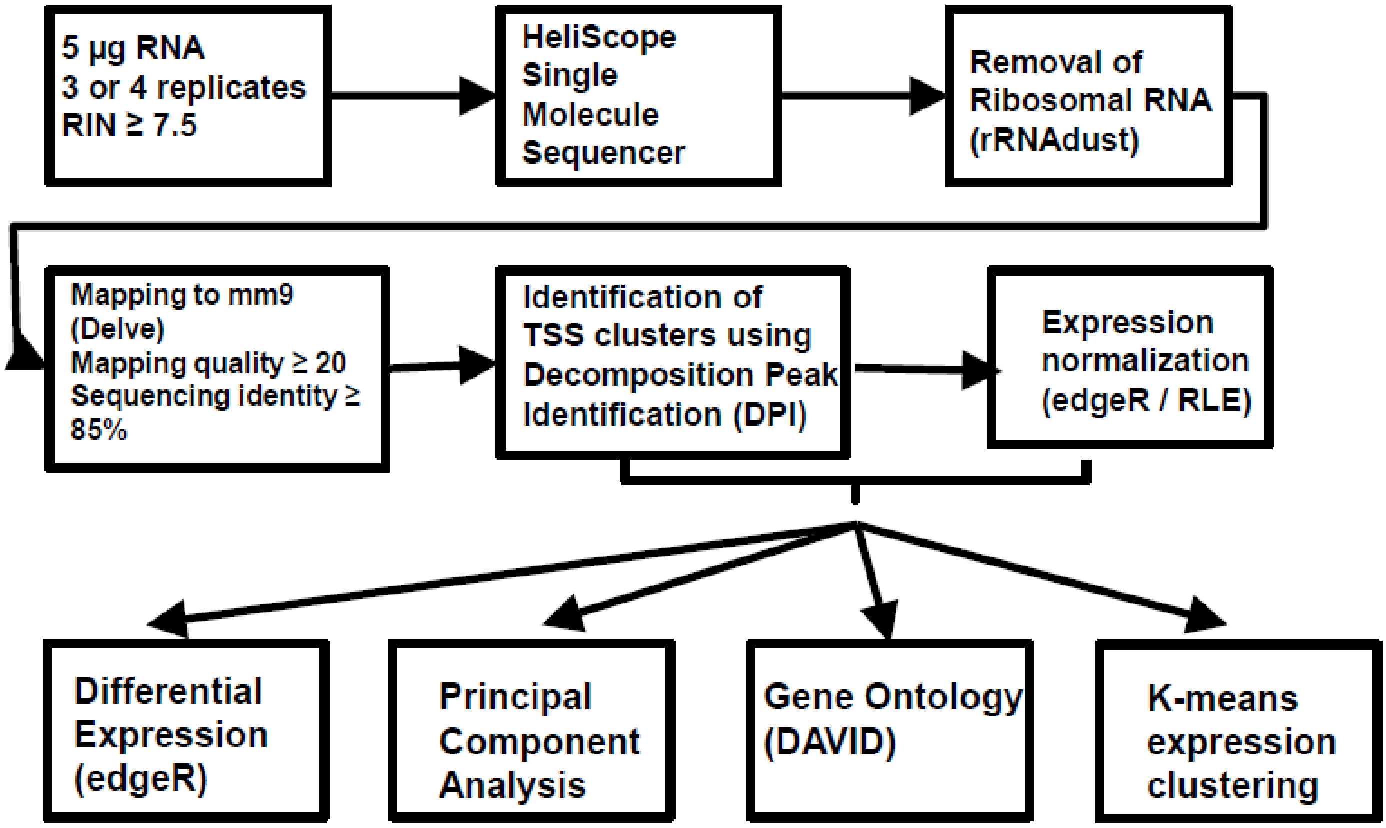

Supplementary Fig.S1 schematic representation of the pipeline for CAGE analysis

Supplementary Fig.S2

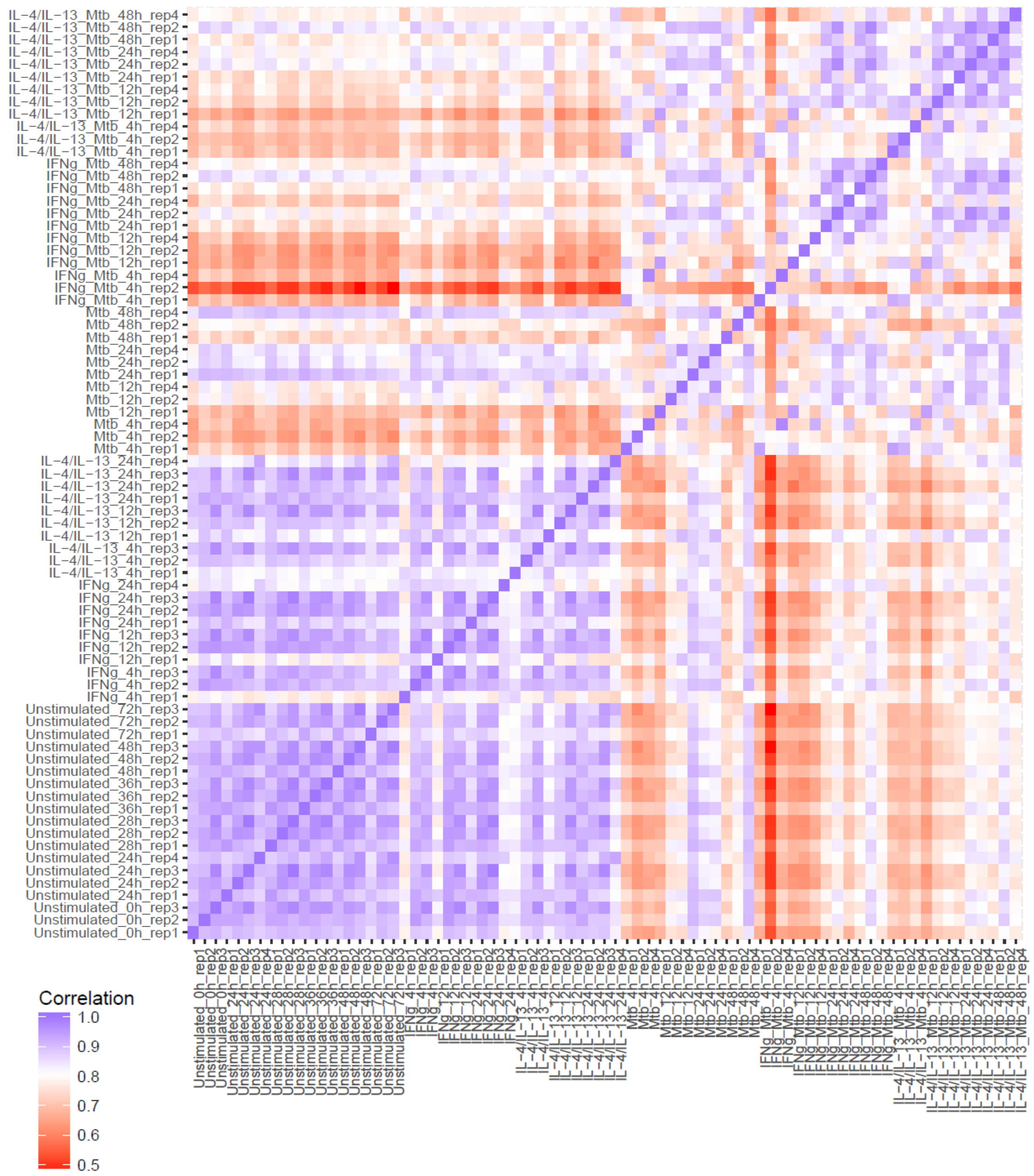

Supplementary Fig.S2 Correlation among biological replicates in CAGE analysis All the biological replicates at each time points were plotted in a correlation heatmap.

Supplementary Fig.S3

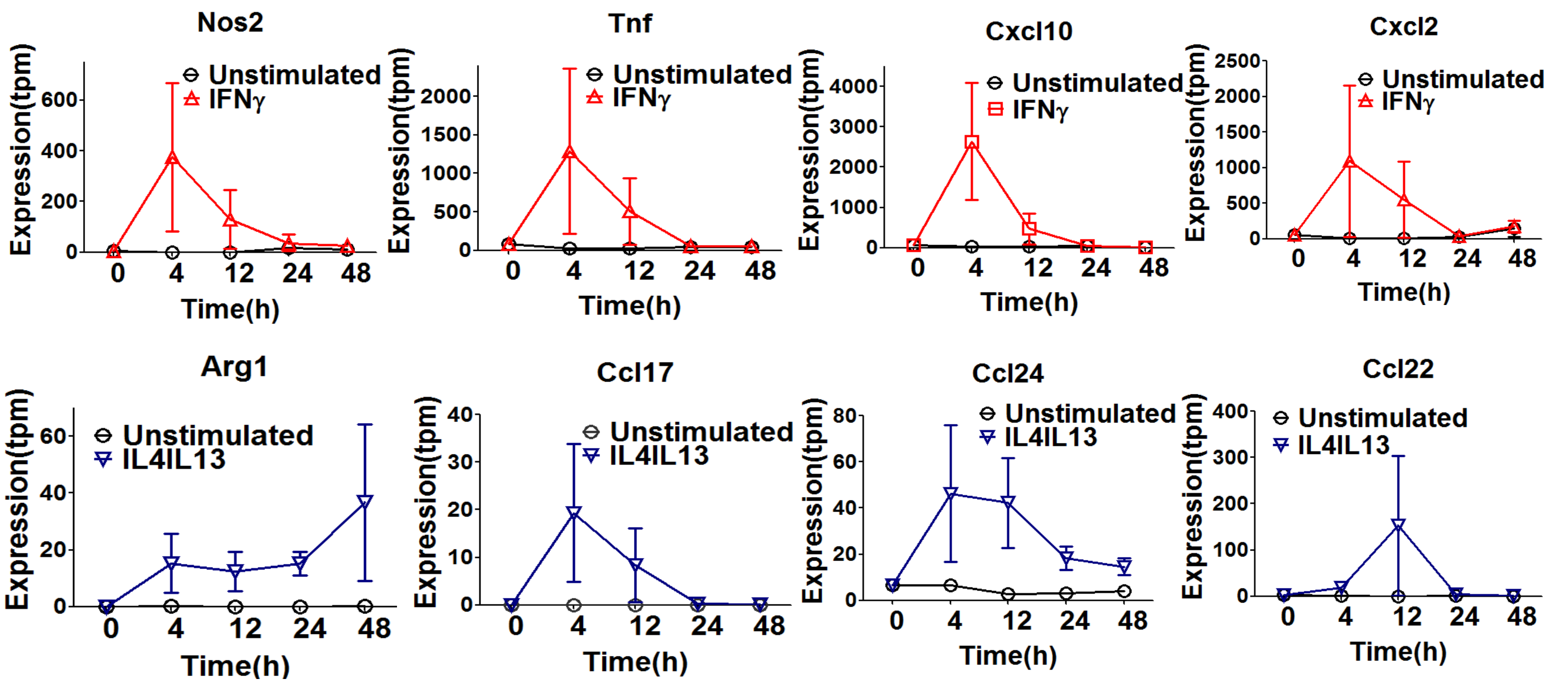

Supplementary Fig.S3 Expression profile of typical M1 and M2 activation marker gene Gene expression profiles of IFNγ-stimulated typical M1 marker genes,*Nos2*, *Tnf*, *Cxcl10* and *Cxcl2* and of IL-4/IL-13-stimulated typical M2marker genes,*Arg1*, *Ccl17*, *Ccl24*and *Ccl22*. The data obtained from three biological experiments was plotted as mean expression and is represented by Tags per Million (TPM).

Supplementary Fig.S4

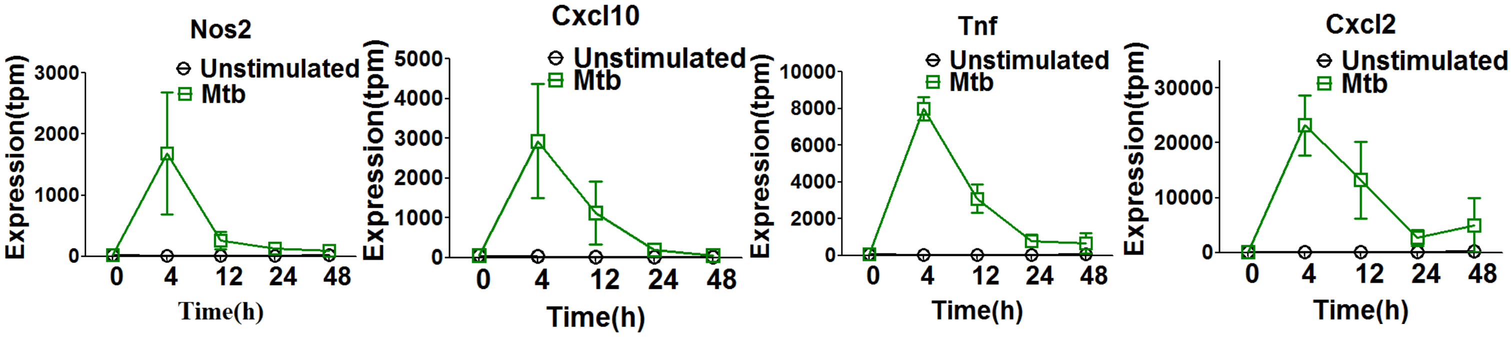

Supplementary Fig.S4Expression profile of typical marker genes of Mtb infected macrophages. The data obtained from three biological experiments was plotted as mean expression and is represented by Tags per Million (TPM).

Supplementary Fig. S5

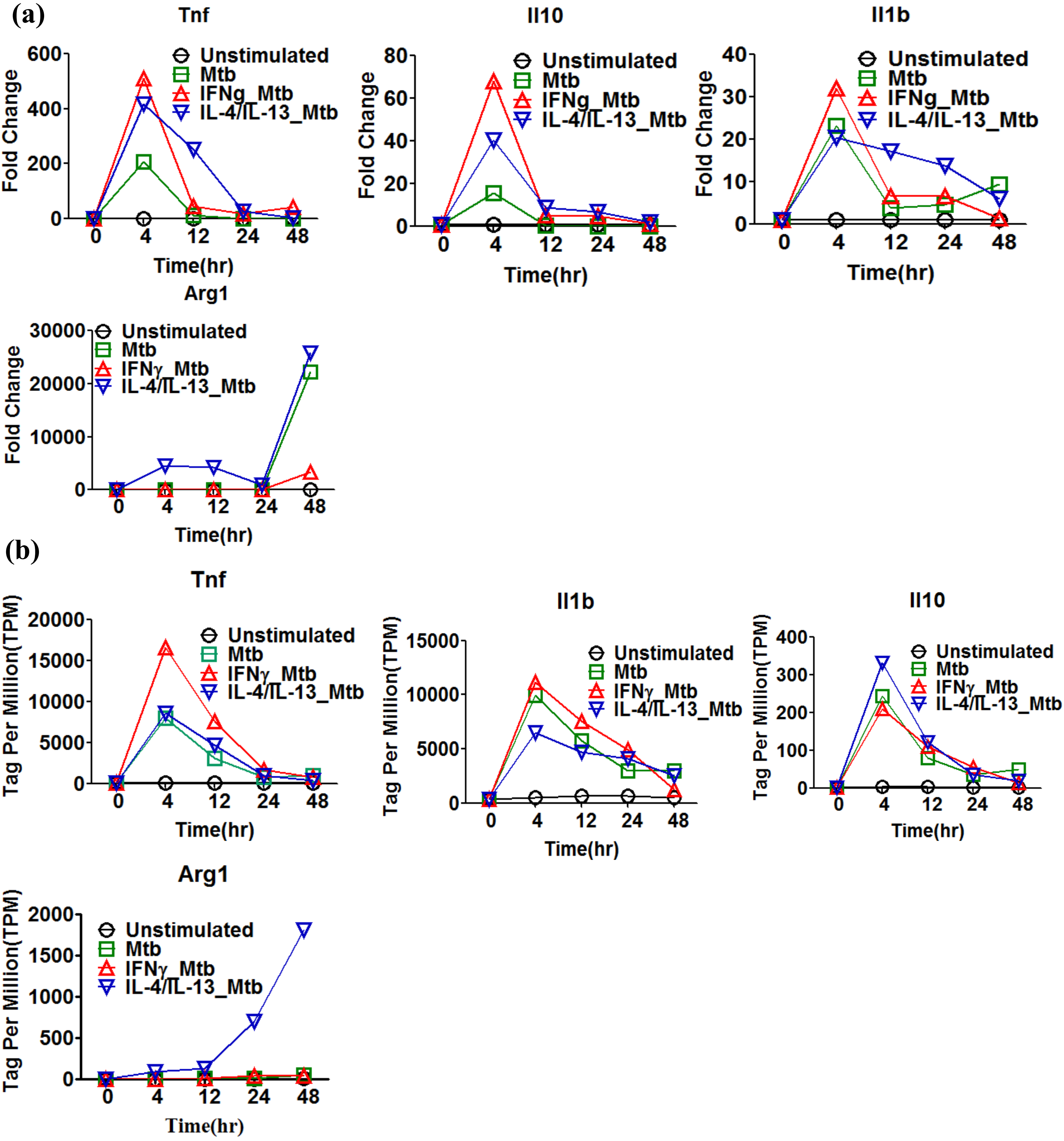

Supplementary Fig.S5. Conformation of CAGE data by qRT PCR. (a) Expression profile of typical marker genes such as *Tnf*, *Il10*, *Il1b*,*Arg1* by q RT PCR. (b) Expression profile of same marker genesby CAGE.

Supplementary Fig.S6

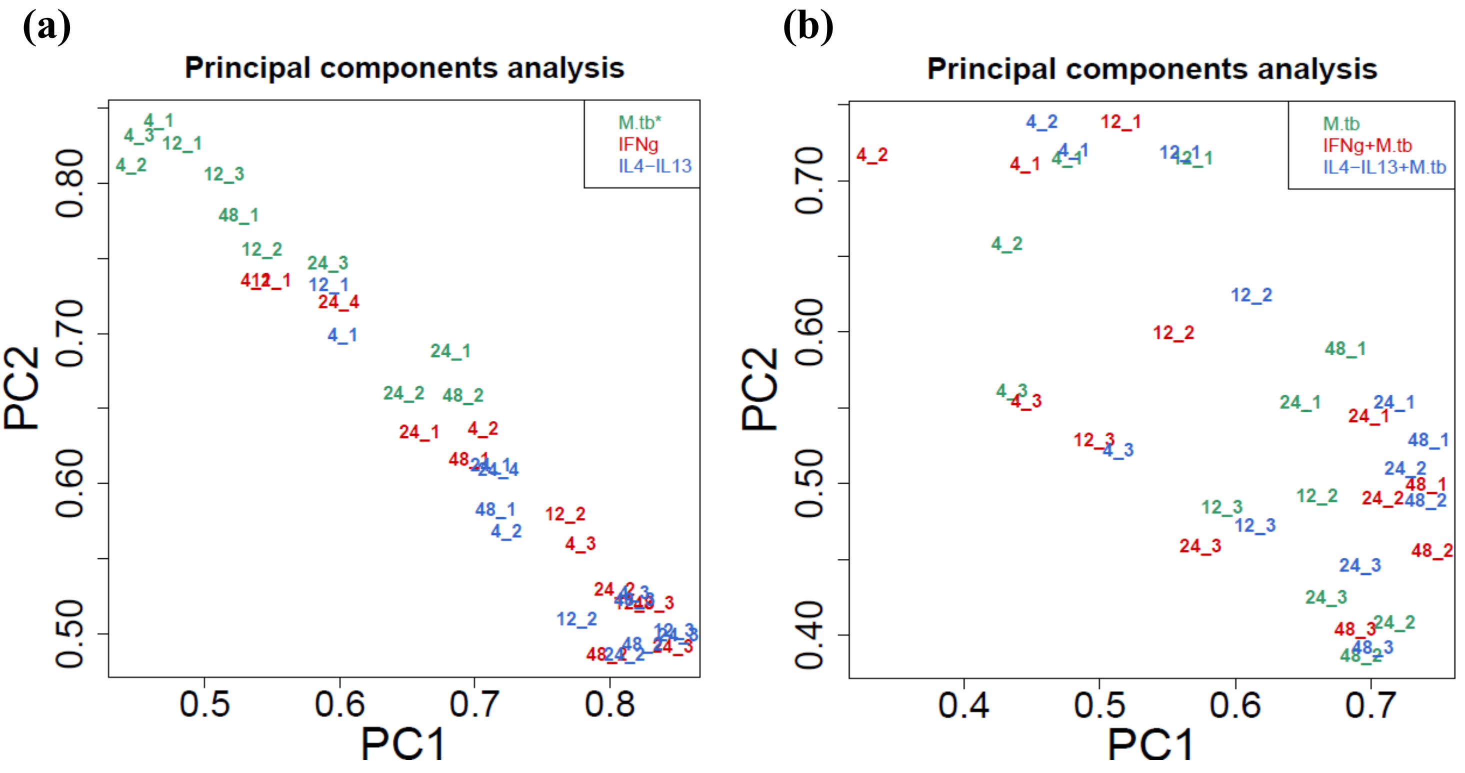

Supplementary Fig.S6 Principal component analysis (PCA). (a) Principal component analysis was performed for IFNγ, IL-4/IL-13, Mtb. Three replicates data, indicated as 1, 2, 3, is shown for the IFNγ, IL-4/IL-13, Mtb. Each treatment has separate colour as indicated in the plot. **(b)**Principal component analysis for IFNγ_Mtb, IL-4/IL-13_Mtb, Mtb. Three replicates data was shown as 1, 2, 3.Each treatment has separate colour ass indicated in the plot.

Supplementary Fig. S7

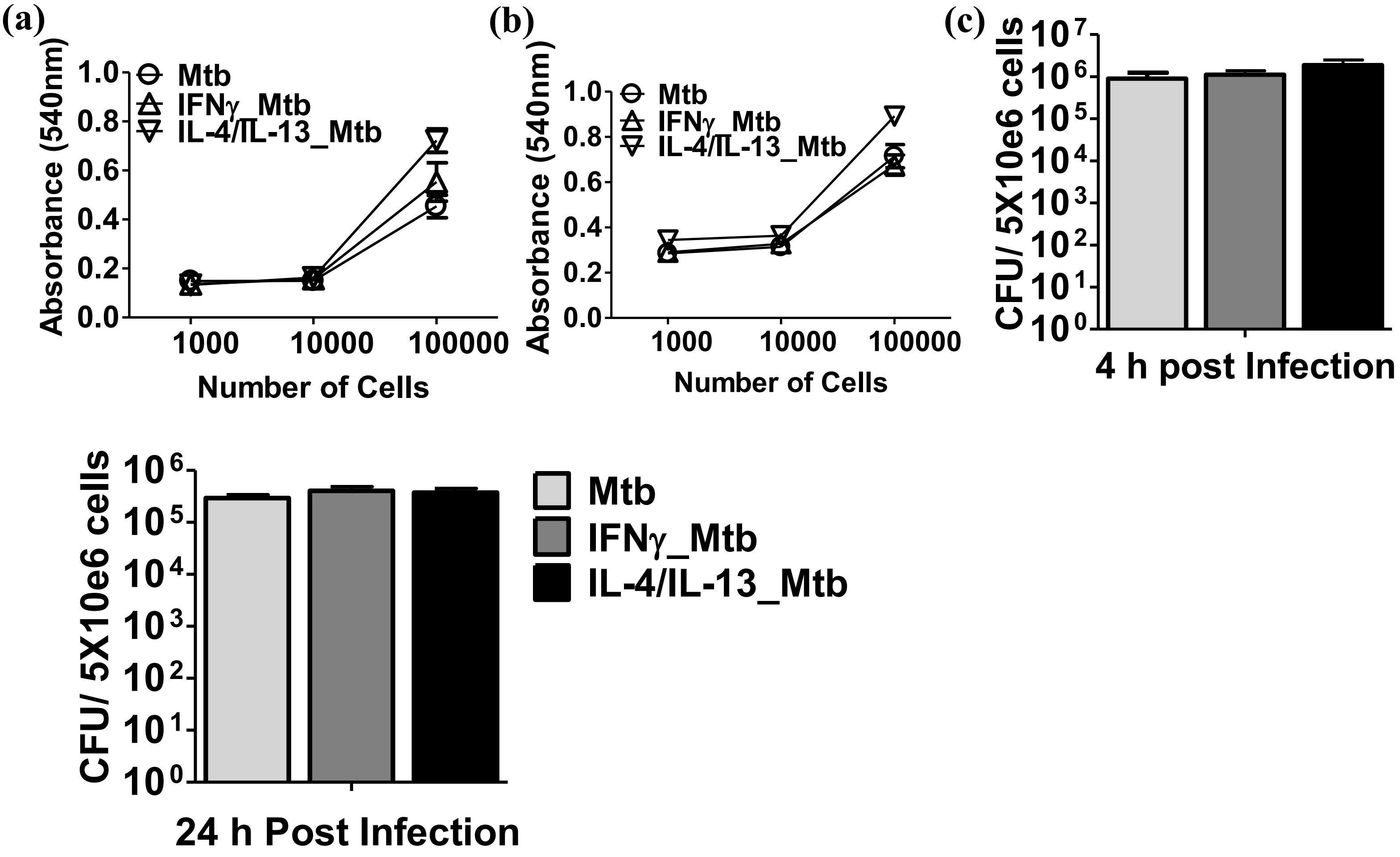

Supplementary Fig. S7(a) and (b) Measurement of cell viability BMDM (1000, 10000, 100000 cell/well in triplicate) cells were either left untreated or stimulated with cytokine IFNγ or IL-4/IL-13 and incubated for 24h. After 24h pre-stimulated and untreated cells were infected with *Mycobacterium tuberculosis* HN878 (MOI=5) for 4 hours. At 4 hours cells was washed to remove the extracellular mycobacteria and replenished with fresh medium containing cytokine (IFNγ or IL-4/IL-13) and gentamycin. At 24h **(a)** and 48h **(b)** post infection MTT was added, absorbance was measure at 540nm

(c) Measurement of colony forming unite (CFU). BMDM (5x 10e6 cells) were either left untreated or stimulated with cytokine IFNγ or IL-4/IL-13 and incubated for 24h. After 24h pre-stimulated and untreated cells were infected with *Mycobacterium tuberculosis* HN878 (MOI=5) for 4 hours. At 4 hours cells was washed to remove the extracellular mycobacteria and replenished with fresh medium containing cytokine (IFNγ or IL-4/IL-13) and gentamycin. The Mtb infected BMDMs were lyzed in 0.1% Triton X100 at 4h and 24h post infection. Cell lysate was used for measurement of CFU by diuting and followed by plateing them in 7H10 agar medium. After 3-4 weeks number of single isolated colony was counted in Mtb, IFNγ_Mtb, IL-4/IL-13_Mtb.

Supplementary Table S5 Number of differentially expressed protein coding genes present in each cluster

Cluster ID	Mtb	IFNγ_Mtb	IL-4/IL-13_Mtb
1	38 (1.6%)	179 (5.7%)	211 (7.2%)
2	365 (15.8%)	560 (17.7%)	658 (22.4%)
3	161 (7.0%)	250 (7.9%)	177 (6.0%)
4	655 (28.5%)	577 (18.2%)	514 (17.5%)
5	176 (7.7%)	224 (7.1%)	212 (7.2%)
6	727 (31.4%)	651 (20.6%)	456 (15.5%)
7	187 (8.0%)	721 (22.8%)	707 (24.1%)

Supplementary Table Legends

Supplementary Table S1 Sample quality assessment RIN scores of RNA samples used for CAGE library preparation and their mapped tags number on genome were shown.

Supplementary Table S2 Differentially expressed protein coding genes (2a) Up- regulated in Mtb infected macrophages (2b) Down – regulated in Mtb infected macrophages (2c) Up and down regulated in IFNγ stimulated macrophages (2d) Up and Down regulated in IL-4/IL-13 stimulated macrophages. Mtb infected macrophages at 4, 12, 24 and 48 hours were compared with non-stimulated non-infected macrophages at 4, 12, 24 and 48 hours to obtain differentially expressed protein coding gene. IFNγ or IL-4/IL-13 stimulated macrophages at 4, 12, 24 and 48 hours were compared with non-stimulated macrophages at 0, 24 and 48 hours to obtain differentially expressed protein coding genes. Protein coding genes, log2 fold-change > 1 in case of up-regulation and log2 fold < -1 in case of down-regulation withsignificant (FDR <0.05) at each time point, were listed up. Fold change are represented by logFC values in which significant values are shown in red and star (*). Expression level was also shown by Tags per million (TPM). One of the column indicates the TF gene. One of the column in table **(2a)** and **(2b)** indicates the K mean cluster number.

Supplementary Table S3 Gene ontology analysis of differentially expressed protein coding genes (3a) and (3b) Up – and down -regulated in Mtb infected macrophages (3c) Up- regulated in IFNγ stimulated macrophages (3d) Up- and down- regulated specifically in IFNγ pre-stimulated Mtb infected macrophages (IFNγ_Mtb) (3E) Up- and down – regulated specifically in IL-4/IL-13 pre-stimulated Mtb Infected macrophages (IL-4/IL-13_Mtb). Gene ontology analysis were performed using differentially expressed, significantly up-and down- regulated protein coding gene list obtained at each time point in Mtb infected macrophages, IFNγ stimulated macrophages using the database for Annotation, Visualization and integrated discovery (DAVID). Gene ontology analysis were performed using differentially expressed, significantly and specifically up- and down- regulated protein coding list obtained at each time point in IFNγ or IL-4/IL-13 stimulated Mtb infected macrophages. Top gene ontology were selected using p value 0.001 and FDR 0.05.

Supplementary Table S4 Differentially expressed protein coding genes (4a) Up- (4b) Down- regulated in IFNγ pre-stimulated Mtb infected macrophages (IFNγ_Mtb) (4c) Up- (4d) Down- regulated in IL-4/IL-13 pre-stimulated Mtb infected macrophages (IL-4/IL-13_Mtb). IFNγ_Mtb and IL-4/IL-13_Mtb infected macrophages at 4, 12, 24 and 48 hours were compared with non-stimulated non-infected macrophages at 4, 12, 24 and 48 hours to obtain differentially expressed protein coding gene. Protein coding genes, log2 fold-change > 1 in case of up-regulation and log2 fold < -1 in case of down-regulation with significant (FDR <0.05) at each time point, were listed up.Fold change are represented by logFC values in which significant values are shown in red and star (*). Expression level was also shown by Tags per million (TPM). One of the column indicates the TF gene. One of the column indicates the K mean cluster number.

Supplementary Table S5. Number of differentially expressed protein coding genes in each cluster. All the 3,805 differentially expressed genes in Mtb, IFNγ_Mtb, IL-4/IL-13_Mtb were subjected to *k*-means clustering analysis. We divided those genes into 7 clusters based on their expression profile. The parenthesis indicates the percentage of genes in each cluster.

Supplementary Table S6 Mtb induced and suppressed gene affected by (6a) and (6b) IFNγ or (6c) and (6d) IL-4/IL-13 pre-stimulation. Several fold elevated or suppressed gene was identified by calculating TMP expression fold change of IFNγ_Mtb in comparison with Mtb, IL-4/IL-13_Mtb samples at each time point. IFNγ pre-stimulation-mediated several fold up regulated **(6a)** and suppressed or down regulated **(6b)** non TF genes (more and less than 10-fold respectively) and TF genes (more and less than 3-fold respectively) was selected at each time point.Qualitative analysis was performed to obtain several fold elevated or suppressed gene in IL-4/IL-13_Mtb by calculating TPM expression fold change comparing expression TPM value of IL-4/IL-13_Mtb with Mtb, IFNγ_Mtb samples at each time point. IL-4/IL-13pre-stimulation-mediated several fold up regulated **(6c)** and suppressed or down regulated **(6d)** non TF genes (more and less than 10-fold respectively) and TF genes (more and less than 3-fold respectively) was selected at each time point.

Supplementary Table S7 Differentially expressed non coding genes (7a) Up- and down- regulated in Mtb infected macrophages (Mtb) (7b) Up- and down- regulated in IFNγ pre-stimulated Mtb infected

macrophages (IFNγ_Mtb) (7c) Up- and down- regulated in IL-4/IL-13 pre-stimulated Mtb infected macrophages (IL-4/IL-13_Mtb). Mtb, IFNγ_Mtb and IL-4/IL-13_Mtb samples at 4, 12, 24 and 48 hours were compared with non-stimulated non-infected macrophages at 4, 12, 24 and 48 hours to obtain differentially expressed lnc RNA gene. Lnc RNA genes, log2 fold-change > 1 in case of up-regulation and log2 fold < -1 in case of down-regulation with significant (FDR <0.05) at each time point, were listed up.Fold change are represented by logFC values in which significant values are shown in red and star (*). Expression level was also shown by Tags per million (TPM). One of the column indicates the K mean cluster number. Last three columns indicate the nearest protein coding gene and their name and their distance from the lnc RNA gene. Second last column indicates the positive and significant (red colour and star *) correlation among the lncRNA and nearest protein coding gene. Last column indicates the "Functional relevance in disease" for differentially expressed lncRNAs in Mtb, IFNγ_Mtb and IL4/IL13_Mtb. In this column, we have provided Pubmed ID of a particular study in which the proximal gene appears to be part of TB signature in these studies. TB signature from these studies are determined either if it is reported in the study or by GEO2R analysis with adjusted p-value < 0.01 and logFC>1 or logFC<-1 if it is not reported in the original study. We have checked eight different publicly available data sets from GEO database or literature. **Supplementary Table S7d** we provide the total proximal genes that are mentioned in Supplementary Table S7a, S7b and S7c in the first column. The second column shows the homologous human gene IDs of these mouse genes from biodb.net. The third column shows the human gene symbols of these human genes from biodb.net. If biodb database does not provide the homologous gene IDs, we checked through Ensembl database whether specific mouse gene have conserved sequence block in human genome and provided that conserved human gene as a homolog of the mouse gene. The remaining columns show the TB transcriptomics studies (Pubmed IDs of articles) which TB signatures were extracted from. The gene list provided in each column for these studies show overlapping genes from particular TB signature and nearest protein coding genes mentioned in Supplementary Table 7a, 7b and 7c.
